# Different Forms of the Adaptogen *Bacopa monnieri (Brahmi)* in the Synthesis of RPU/PIR Foams

**DOI:** 10.3390/polym18121471

**Published:** 2026-06-11

**Authors:** Joanna Liszkowska, Justyna Miłek, Krzysztof Moraczewski, Krzysztof Szabliński

**Affiliations:** 1 Department of Chemistry and Technology of Polyurethanes, Faculty of Materials Engineering, Kazimierz Wielki University, J. K. Chodkiewicza 30, PL 85-064 Bydgoszcz, Poland; 2 Department of Chemical and Biochemical Engineering, Faculty of Chemical Technology and Engineering, Bydgoszcz University of Science and Technology, Seminaryjna 3, 85-326 Bydgoszcz, Poland; jmilek@pbs.edu.pl; 3 Department of Polymer Materials Engineering, Faculty of Materials Engineering, Kazimierz Wielki University, J. K. Chodkiewicza 30, PL 85-064 Bydgoszcz, Poland; kmm@ukw.edu.pl (K.M.); k.sza@ukw.edu.pl (K.S.)

**Keywords:** adaptogen, antioxidant, *Bacopa monnieri*, polyurethane foams, thermal properties, structure, DSC

## Abstract

Various forms of *Bacopa monnieri* (BM), including original powder (Mp), tea form (Mo), and post-extraction residues (Mf), were used as natural bio-based additives in rigid polyurethane–polyisocyanurate (RPU/PIR) foams. The study investigated the influence of BM form and content on the physical, mechanical, thermal, and flammability properties of the foams. The results demonstrated that both the type and concentration of BM significantly affected foam performance. Foams containing Mf exhibited the lowest apparent density and reduced brittleness, whereas foams modified with Mp showed the highest compressive strength. The incorporation of BM also contributed to reduced flammability and enhanced thermal resistance of the foams. Thermal analysis indicated that BM additives modified the degradation behavior of RPU/PIR foams by promoting char formation and improving thermal stability at elevated temperatures. In particular, samples containing tea and post-extraction residues showed increased stability of the carbonized residue during the final degradation stage. The most favorable overall properties were obtained for BM contents between 3 and 7 wt%, while higher filler concentrations negatively affected the structural integrity of the foam matrix. The results confirm that the performance of RPU/PIR foams strongly depends on the balance between matrix continuity and biofiller functionality. The obtained materials show potential for application in floristry products and lightweight insulating systems where low density, dimensional stability, and enhanced thermal resistance are required.

## 1. Summary

Adaptogens are plant-derived bioactive compounds that support the organism’s resistance to physical, chemical, and environmental stressors through modulation of physiological homeostasis [1,2]. Among them, *Bacopa monnieri* is particularly rich in polyphenols, flavonoids, saponins, and other antioxidant compounds exhibiting radical-scavenging activity [3,4]. Similar antioxidant-rich plant materials, including green tea, turmeric, grapes, Schisandra chinensis, and cocoa, contain phenolic compounds capable of reducing oxidative stress and stabilizing reactive oxygen species [5,6,7,8].

Plant polyphenols constitute an important group of secondary metabolites responsible for antioxidant activity in natural systems. Their structure, typically containing multiple phenolic hydroxyl groups, enables free radical neutralization and inhibition of oxidative reactions [9,10,11]. Phenolic compounds include phenolic acids, flavonoids, lignans, tannins, and other aromatic derivatives synthesized by plants as protective agents against environmental stress factors such as UV radiation and oxidation [12,13]. Due to these properties, natural polyphenols are increasingly investigated as bio-based stabilizing additives for polymeric materials.

Polymeric materials, including polyurethane (PU) systems, are highly susceptible to thermo-oxidative degradation during processing and long-term use [14]. Exposure to elevated temperature, oxygen, UV radiation, humidity, and atmospheric pollutants leads to chain scission, discoloration, embrittlement, and deterioration of mechanical properties [15,16,17]. In rigid polyurethane–polyisocyanurate (RPU/PIR) foams, excessive thermal exposure may additionally induce structural degradation and partial carbonization of the foam core. Furthermore, prolonged environmental exposure accelerates oxidative degradation processes, particularly in the presence of nitrogen oxides and reactive oxygen species [18].

To improve thermal and oxidative stability, various antioxidants and stabilizers are commonly introduced into PU systems. Conventional stabilizers include hindered phenols, phosphites, hydroxylamines, and hindered amine light stabilizers (HALSs) [19,20,21]. Recently, increasing attention has been devoted to natural bio-based antioxidants due to their renewable origin, environmental compatibility, and potential multifunctional behavior [22,23]. Natural fillers rich in polyphenolic compounds may additionally promote char formation during thermal degradation, improving flame resistance and thermal stability of polymer composites [24,25].

Commercial floral foams are commonly based on phenol–formaldehyde resins and are designed to combine high water absorption, open-cell morphology, low density, and sufficient mechanical stability to support flower stems. However, conventional floral foams are petroleum-derived, non-biodegradable materials that generate microplastic particles during use and disposal. Recent studies highlighted their environmental impact and potential toxicity toward aquatic organisms. Therefore, there is increasing interest in developing sustainable and partially biodegradable alternatives for floristry applications. In this context, the material developed in this work demonstrates promising characteristics such as high water uptake, porous structure, mechanical integrity, and biodegradability, which are important functional requirements for floral foam applications [26,27,28,29].

Despite growing interest in bio-based additives, the application of *Bacopa monnieri* in polyurethane systems remains largely unexplored. Most previous studies concerning this plant focused on its pharmacological and antioxidant properties rather than its potential role as a functional modifier in polymer materials. Therefore, the novelty of the present study lies in the use of three different forms of *Bacopa monnieri,* namely, powder (Mp), tea form (Mo), and post-extraction residues (Mf), as multifunctional bio-based additives for rigid polyurethane–polyisocyanurate foams.

The aim of this study was to evaluate the influence of different forms and contents of *Bacopa monnieri* on the structure, thermal stability, flammability, mechanical performance, and degradation behavior of RPU/PIR foams. Particular attention was devoted to the effect of accelerated aging conditions involving UV radiation, temperature, and humidity. The study also investigated the potential application of post-extraction *Bacopa monnieri* residues as a waste-derived functional filler in lightweight foam materials intended for floristry applications.

## 2. Materials

The ratio of isocyanate to polyol, as well as the type of catalysts and blowing agents used, have a significant impact on foam properties and, consequently, on their potential applications and usability [30,31,32,33]. Raw materials determine the chemical and cellular structure of the product, which in turn affects, among other aspects, thermal and flame resistance, as well as stability and mechanical strength.

**Figure 1:**
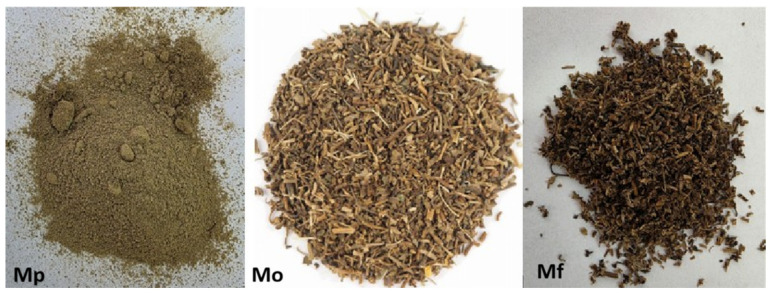
Fillers *Bacopa monnieri* (BM) in various forms: Mp—powder, Mo—dried tea, Mf—grounds (waste after brewing Mo).

In the synthesis of the polymer foam materials (RPU-PIR) containing plant raw material (*Bacopa monnieri*), the following were used: Rokopol RF551 polyol (66.8 g) and Silicon Genapol X080 (5.4 g). The catalytic system in the RPU/PIR formulation included a 33% solution of anhydrous potassium acetate (produced by Chempur, Piekary Śląskie, Poland) in diethylene glycol (produced by Chempur, Poland) as a trimerization catalyst and a 33% solution of DABCO (1.4-diazabicyclo[2.2.2]octane, produced by Alfa Aesar, Haverhill, MA, USA) in diethylene glycol as a polyurethane bond catalyst. The stabilizer used for the foam structure was Genapol X 080—a fatty alcohol polyglycol ether (Clariant Producte (Deutschland) GmbH, Brzeg Dolny, 65926 Frankfurt am Main). Carbon dioxide produced in situ in the reaction between water and isocyanate groups was a blowing agent. As a flame retardant, we used Roflam F5—phenol isopropylated phosphate; the density at 25 °C was 1.15–1.25, and the dynamic viscosity at 25 °C was 48–67 mPas (supplied by PCC Rokita S.A., Poland). Moreover, the following were added: water (3.15 g) and Purocyn B isocyanate (250.7 g). The origin and description of the raw materials used for the synthesis of foam M0 were presented in previous studies [34,35,36]. Additionally, *Bacopa monnieri* (Figure 1) was added in three forms: original green powder (Mp), dried unbrewed tea form (Mo), and spent residues (Mf) obtained after brewing Mo as waste material(grounds). The spent residue is a material theoretically depleted of part of the extractives and low-molecular-weight compounds, more fibrous in nature (e.g., cellulose and lignin) and chemically poorer. *Bacopa monnieri* originates from India (Sattva Group Bombay Bazaar). Each form of *Bacopa monnieri* was added in amounts of 1 wt% (3.18 g), 3 wt% (9.54 g), 7 wt% (22.26 g), and 13 wt% (41.45 g) relative to the total mass of polyol and polyisocyanate. In this way, three series of foams (Mp, Mo, and Mf) were obtained.

## 3. Methods

The foams were obtained by the block method [37,38]. The foam ingredients (except the isocyanate) were mixed using a 20 cm mechanical stirrer at 1800 rpm until completely mixed. After mixing the components of this polyol premix, the isocyanate was added and mixed for an additional 10 s. The mixture was poured into an open, rectangular mold measuring 190 mm × 190 mm × 230 mm. Each foam was produced three times to ensure repeatability of results. After preparation, the foams were stored for 24 h at room temperature. The resulting foam blocks were cut into test samples with an accuracy of 0.1 mm and weighed with an accuracy of 0.01 g. Five samples were cut from each foam for each test.

The tests were performed according to applicable standards (Table 1). Foams were analyzed for color, density, absorbency and water absorption, compressive strength, structure, thermal properties (DSC and DTA), flammability, and degradation.

All experiments were performed in quintuplicate, and the results are presented as mean values ± standard deviations (SDs). Statistical analysis was carried out using one-way analysis of variance (ANOVA) followed by Tukey’s post hoc test to determine significant differences between samples. Differences were considered statistically significant at *p* < 0.05.

## 4. Results and Discussion

The selection of raw materials has a significant impact on foam properties and, consequently, on their potential applications [39]. Polyurethanes are formed in the reaction between polyols and isocyanates, and their final properties depend not only on the type of these basic substrates but also on additives such as catalysts, blowing agents (porofors), stabilizers, and fillers [40]. Changing the type of raw materials leads to significant differences in the cellular structure of foams (cell size and distribution and the proportion of closed/open cells), which directly affects their mechanical, thermal, and functional properties.

The use of different blowing agents influences the foaming process and the cellular structure, and thus the density and strength of the foams [40]. The type of polyol affects the rigidity and thermal stability of the material [41]. Additives (e.g., fillers, fibers, and solid particles) can increase strength, modify brittleness, or improve heat resistance [42].

### 4.1. Foam Processing

The synthesis parameters of RPU/PIR foams (rigid polyurethane and polyisocyanurate foams) are of crucial importance, as they directly determine their chemical and cellular structure and consequently their functional properties and range of applications. Processing times depend on the raw materials used [30,31,32,33,39,40]. Processing conditions such as temperature, mixing speed, and other process parameters may influence the performance of the surfactant, which in turn affects the final foam properties.

The processing parameters of foams modified with different forms of Bacopa monnieri are presented in Table 2. The results show that the Mf series is the most reactive system, as compared to Mp and Mo, exhibiting the shortest processing times (e.g., cream time: 11–13 s, string time: 21–40 s, tack free time: 23–47 s). An increase in the content of Bacopa monnieri leads to an extension of all processing parameters. Despite the presence of the filler, the Mf system still maintains relatively high reactivity compared to the Mp and Mo series. The observed changes are mainly attributed to an increase in the viscosity and density of the polyol premix with the rising content of solid fractions of *Bacopa monnieri*, which limits the mobility of reactants and consequently slows down the foam formation process.

The foam blowing reaction is exothermic. The process temperature ranges from 174 °C to 178 °C. No linear relationship was observed between the amount and type of filler and the maximum reaction temperature (T_max_). Only a slight increase in Tmax was noted with the increasing content of Mp, Mo, or Mf in the foam, by approximately 3–7 °C compared to the reference foam, M0. *Bacopa monnieri* does not significantly affect the intensity of the exothermic reaction but mainly modifies the process kinetics. It acts primarily as a rheological modifier of the polyol system.

### 4.2. Foam Structure

The foam structure is formed as a result of an increase in the number or the size of gas bubbles within the polymer matrix. Initially, the foam density decreases slightly, and small, dispersed spherical gas bubbles form in the liquid matrix. In the next stage, the cells become closed. Subsequently, the rupture of the cell walls leads to their opening. The ratio of open to closed cells is crucial for the properties of the foams. Pore size is also of particular importance for the final properties of the material [43,44].

#### 4.2.1. Anisotropy

The study of foam structure includes the measurement of cells both along and opposite to the direction of foam rise, before degradation and after environmental degradation.

**Figure 2:**
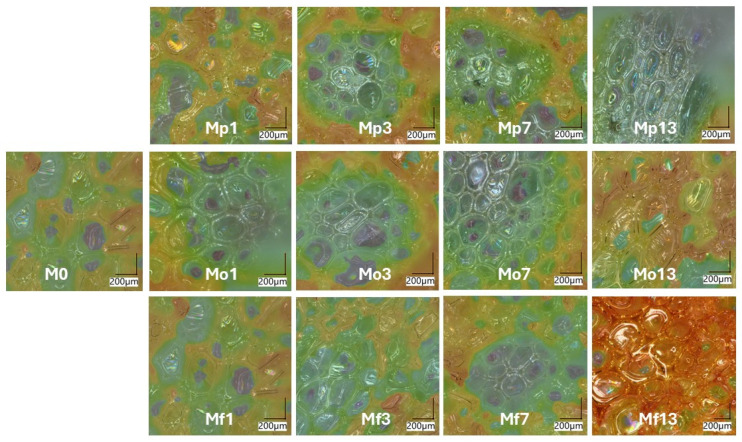
Study of cell structure before degradation, against the direction of growth.

**Figure 3:**
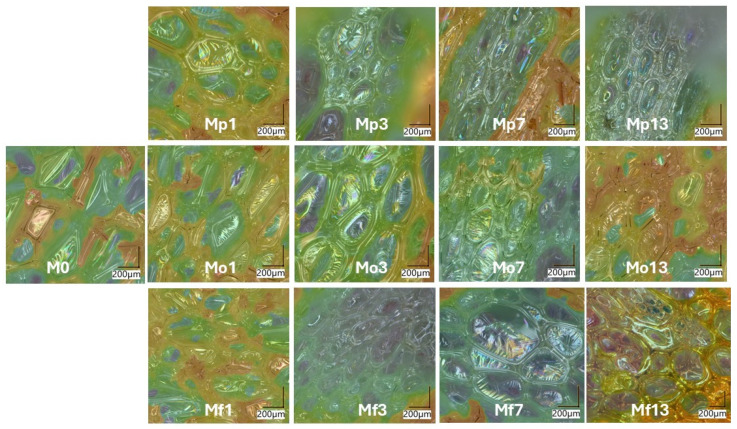
Study of cell structure before degradation, in the direction of growth.

**Figure 4:**
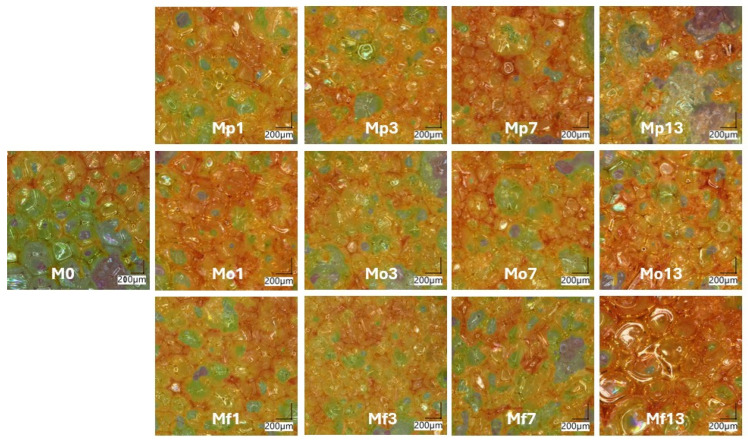
Study of cell structure after degradation, against the direction of growth.

**Figure 5:**
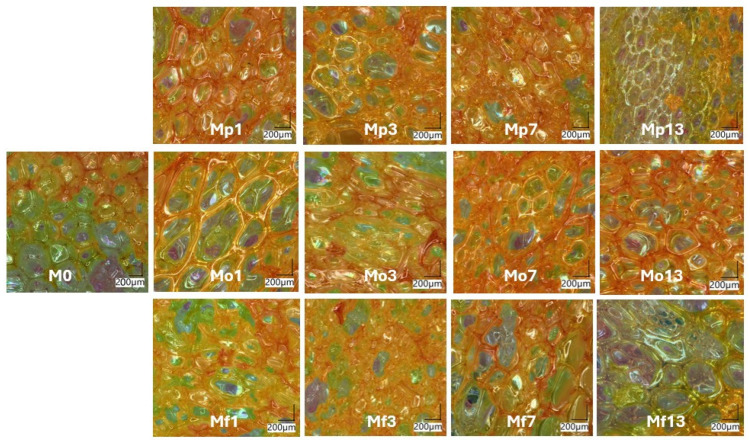
Study of cell structure after degradation, in the direction of growth.

Based on microscope images (Figure 2, Figure 3, Figure 4 and Figure 5), the width and height of foam cells were determined (Table 3). Subsequently, their anisotropy was calculated as the ratio of width to height. In Table 3, the letter “p” next to the foam symbol indicates measurements performed on samples cut perpendicular to the direction of foam rise, while the letter “z” indicates samples cut parallel to the direction of foam rise. The letter “D” denotes degraded foams. An anisotropy value equal to 1 indicates a perfectly spherical cell. Values below 1 indicate elongation in the vertical direction (along the foam rise direction), whereas values above 1 indicate elongation in the horizontal direction (cell width).

Microscopic images taken before degradation along the foam rise direction (Figure 3 and Figure 4) show that the cells are elongated compared to those observed in samples cut perpendicular to the rise direction, which exhibit a more spherical shape. Based on these observations, it can be concluded that cell anisotropy differs from 1, which was confirmed by measurements (Table 3). The cells in foams containing Mp and Mf fillers decreased in size by approximately half compared to the reference foam, M0, while in the Mo series the reduction was about 30–40%. However, with increasing filler content, the cell size increased. A decrease in anisotropy was also observed for foams marked “z” (measured along the rise direction), with an average anisotropy of approximately 0.6, compared to foams marked “p”, where the average anisotropy was about 0.8, indicating cell elongation.

The reason for cell elongation was the increase in processing times with rising filler content.

Foam degradation did not significantly affect cell size in the degraded (D series) foams compared to the non-degraded samples. However, foams containing 13 wt% filler exhibited a high variability in cell size distribution, including the presence of large, medium, and small cells. Therefore, the calculated average values of cell width and height for these samples represent heterogeneous cellular structures. The increased cell size heterogeneity at high filler loading suggests that excessive additive content adversely affects the uniformity of foam morphology.

#### 4.2.2. Degraded Layer in RPU/PIR

**Figure 6:**
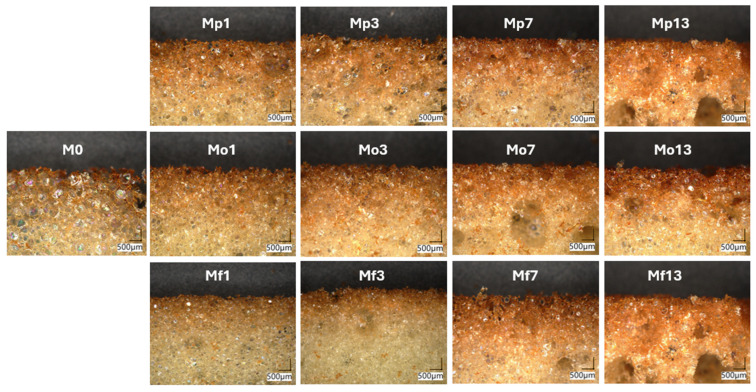
Study of degraded layer of foams.

**Figure 7:**
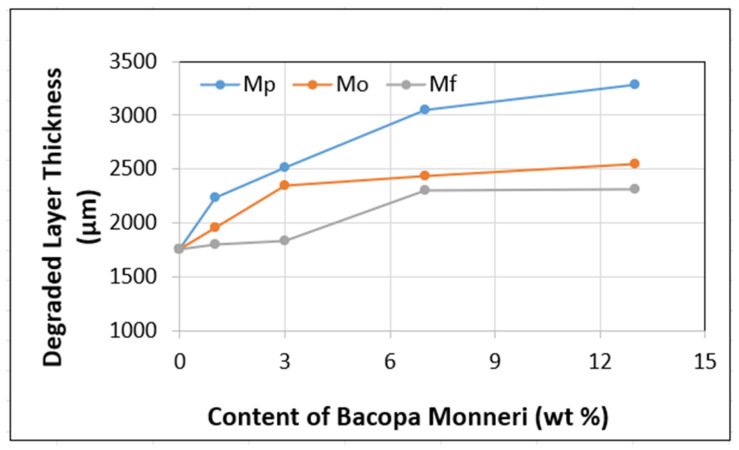
Dependence of degraded layers of foams on *Bacopa monnieri* content in foam.

The thickness of the degraded layer in RPU/PIR foams was found to strongly depend on both the content and the form of the *Bacopa monnieri* filler (Figure 6). As shown in Figure 7, an overall increase in degraded layer thickness can be observed with increasing filler content for all investigated systems; however, the magnitude of this effect varies significantly depending on the type of additive.

For foams modified with *Bacopa monnieri* powder (Mp), the highest degradation depths were recorded across the entire composition range. The degraded layer thickness increased markedly from approximately 2200 µm at 1 wt% to about 3282 µm at 13 wt%, indicating a strong promotion of degradation processes. This behavior suggests that the powdered form of the additive facilitates the penetration of degrading factors (e.g., UV radiation and moisture) into the foam structure and may introduce structural heterogeneities that act as initiation sites for degradation.

In the case of foams containing *Bacopa monnieri* in the form of tea (Mo), intermediate values of degraded layer thickness were observed. The thickness increased from approximately 1950 µm at 1 wt% to around 2543 µm at 13 wt%, with a tendency toward gradual saturation at higher filler contents. This indicates a more balanced effect, where the presence of bioactive compounds may partially counteract degradation, while the plant-based structure still contributes to the formation of pathways facilitating environmental attack.

Foams modified with spent *Bacopa monnieri* residues (Mf) exhibited the lowest degradation depths. The degraded layer thickness increased only slightly from approximately 1800 µm at 1 wt% to about 2314 µm at 13 wt%, with a clear plateau at higher filler contents. This behavior suggests that the extracted plant material acts primarily as a physical barrier, limiting the diffusion of degrading agents into the polymer matrix. The reduced content of reactive compounds in the spent material likely contributes to this stabilizing effect.

The more pronounced degradation observed for Mp-modified foams compared to Mf systems can be attributed to differences in the chemical composition and physical structure of the fillers. The Mp fraction contains a higher proportion of extractive compounds and low-molecular-weight components, which may act as reactive sites and facilitate photo-oxidative and hydrolytic degradation of the polymer matrix. In contrast, the Mf fraction is enriched in lignocellulosic structures (cellulose and lignin), which are more thermally stable and tend to promote char formation, thereby acting as a partial protective barrier against further degradation. As a result, Mp accelerates the degradation process by increasing the availability of reactive species, whereas Mf contributes more to structural stabilization through carbonaceous residue formation.

The results indicate that while increasing filler content generally promotes deeper degradation, the form of *Bacopa monnieri* plays a decisive role in controlling the degradation mechanism. The powdered form enhances bulk degradation, the tea form exhibits an intermediate effect, and the spent residues effectively limit the depth of degradation. These findings highlight the importance of filler processing in tailoring the durability of RPU/PIR foams under environmental exposure.

### 4.3. Color Change Analysis of Foams

**Figure 8:**
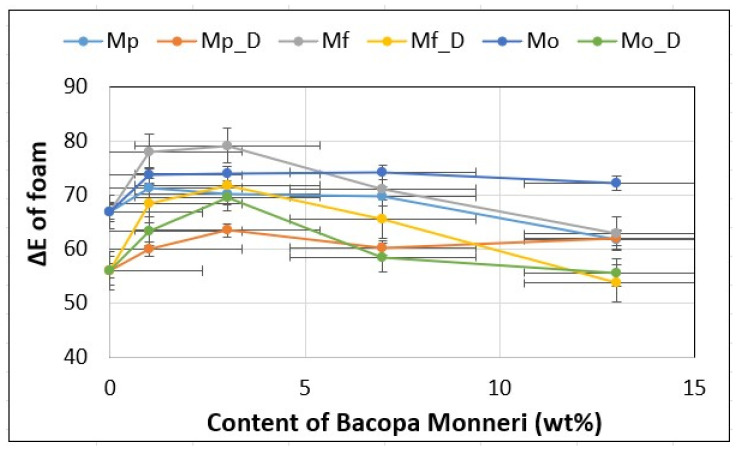
Dependence of ΔE on *Bacopa monnieri* content in foam.

The color change (ΔE) of RPU/PIR foams was significantly affected by both the incorporation of *Bacopa monnieri* fillers and environmental degradation (Figure 8). In non-degraded systems, all modified foams exhibited higher ΔE values compared to the reference sample, confirming that the addition of plant-based fillers inherently modifies the optical properties of the polyurethane matrix. The most pronounced effect was observed for foams containing spent residues (Mf), where ΔE values reached approximately 75–80 at low and moderate filler contents. This strong color variation can be attributed to the high content of lignocellulosic components (cellulose, hemicellulose, and lignin), which introduce intrinsic chromophoric groups and increase light absorption. Mp and Mo systems showed slightly lower but still elevated ΔE values (approximately 68–74), indicating a less intensive but still significant contribution of the filler to color formation.

Environmental degradation induced clear changes in ΔE behavior, which depended strongly on the filler form. In Mp_D systems, ΔE values remained relatively stable or slightly decreased with increasing filler content (approximately 61–62 at higher loadings), suggesting partial color homogenization. This effect may be associated with surface oxidation processes and the formation of more uniform chromophoric structures during UV- and thermo-oxidative aging.

A more pronounced decrease in ΔE was observed for Mo_D foams, where values gradually decreased from approximately 70 to about 55 with increasing filler content. This trend suggests degradation of thermally and photo-sensitive compounds present in the tea-derived filler, leading to partial fading and reduced color intensity after environmental exposure.

The strongest reduction in ΔE after degradation was observed for Mf_D systems, particularly at higher filler loadings (approximately 53–55). This behavior indicates that extracted plant residues undergo significant structural and chemical transformations during aging, including degradation of lignocellulosic chromophores and increased surface uniformity due to oxidative processes.

Overall, the results demonstrate that environmental degradation tends to reduce color differences in RPU/PIR foams containing Bacopa monnieri, primarily due to oxidation of organic chromophores and structural reorganization of the polymer surface. However, the magnitude of this effect strongly depends on the filler form: Mf systems are the most sensitive to degradation-induced color changes, while Mp systems exhibit the highest color stability, likely due to a lower fraction of easily degradable bioactive compounds and a more chemically stable particulate structure.

Overall, the combined DSC, TGA, color change (ΔE), and microscopy results indicate a coherent degradation mechanism of RPU/PIR foams modified with Bacopa monnieri. Environmental exposure induces initial chain scission and oxidation of the polymer matrix, which is reflected in reduced Δm_1_ values (TGA), broadened thermal transitions (DSC), increased structural heterogeneity (microscopy), and partial transformation of chromophoric structures responsible for color changes (ΔE).

These effects are strongly dependent on the filler form, which governs the balance between degradation promotion (via reactive low-molecular-weight species in Mp systems) and structural stabilization through lignocellulosic char-forming components (Mf systems), ultimately controlling both the thermal and optical stability of the foams.

### 4.4. Absorptivity and Water Absorption

**Figure 9:**
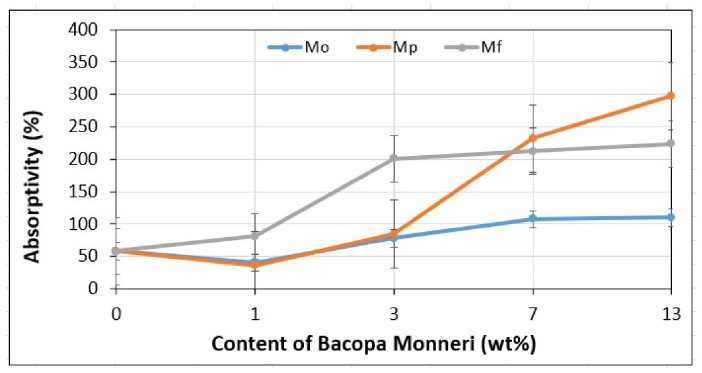
Dependence of absorptivity on *Bacopa monnieri* content in foam.

**Figure 10:**
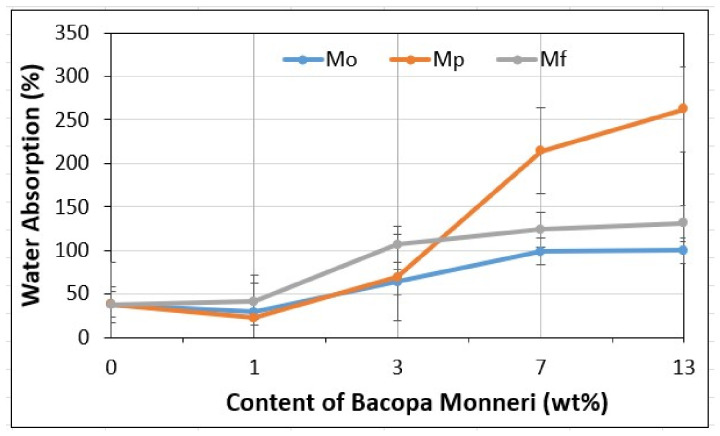
Dependence of water absorption on *Bacopa monnieri* content in foam.

The water-related properties of RPU/PIR foams, including absorptivity (Figure 9 and total water absorption (Figure 10, were strongly influenced by both the content and the form of *Bacopa monnieri*. Although both parameters are associated with water uptake, they describe different aspects of the process: absorptivity reflects the rate and ability of the material to uptake water via capillary action, while water absorption represents the total amount of water retained within the structure.

Absorptivity increased with increasing filler content for all systems; however, the magnitude of this increase strongly depended on the filler form. The most pronounced effect was observed for foams modified with powdered *Bacopa monnieri* (Mp), where absorptivity increased dramatically, reaching approximately 300% at 13 wt%. This indicates a significant enhancement of capillary transport, suggesting the formation of a more open and heterogeneous cellular structure facilitating rapid water ingress. Foams containing spent residues (Mf) exhibited a rapid increase in absorptivity at low and moderate contents, reaching approximately 200% at 3 wt%, followed by a plateau at higher loadings (~220–230%). This behavior indicates that structural changes affecting capillary uptake occur primarily at lower filler contents, after which the system reaches a saturation state. In contrast, foams modified with *Bacopa monnieri* in the form of tea (Mo) showed the lowest absorptivity values and a gradual increase with filler content, reaching approximately 110% at 13 wt%. This suggests a more homogeneous structure with fewer pathways for rapid capillary transport.

The trends observed for total water absorption were consistent with those for absorptivity but less pronounced. Foams containing Mp exhibited the highest increase in water absorption, reaching approximately 260% at 13 wt%, confirming that the structural modifications promoting capillary uptake also lead to increased overall water retention. Mf-modified foams showed moderate water absorption, increasing to approximately 130% at the highest filler content. Although absorptivity reached a plateau at higher contents, total water absorption continued to increase slightly, indicating that while capillary pathways stabilize, the overall pore volume or water retention capacity still increases. Mo systems again exhibited the lowest values, with water absorption reaching approximately 100% at 13 wt%. This confirms that this filler form has the least disruptive effect on the foam structure and limits water uptake.

A clear correlation between absorptivity and water absorption can be observed, indicating that both parameters are governed by similar structural features, such as porosity, cell openness, and filler dispersion. However, differences in the trends highlight the distinct nature of these parameters. Absorptivity is more sensitive to early structural changes and capillary pathways, as evidenced by the rapid increase and plateau observed for Mf systems. In contrast, water absorption reflects the cumulative storage capacity of the foam and continues to increase even when absorptivity stabilizes.

The results demonstrate that the incorporation of *Bacopa monnieri* modifies both the kinetics and capacity of water uptake in RPU/PIR foams. The powdered form (Mp) induces the most significant structural disruption, leading to both high absorptivity and water absorption. The spent residues (Mf) promote rapid capillary uptake at low contents but exhibit saturation behavior at higher loadings. In contrast, the tea form (Mo) results in the lowest water uptake, indicating better structural integrity and reduced permeability.

The very high absorptivity values observed for Mp foams (approaching 300% at 13 wt%) are associated with the formation of a highly open and heterogeneous cellular structure, as confirmed by microscopy analysis. Disrupted cell walls and interconnected pores facilitate rapid capillary water transport throughout the foam volume. Additionally, the hydrophilic nature of lignocellulosic components, rich in hydroxyl-containing cellulose and hemicellulose structures, further enhances water affinity. Similar trends have been reported for polyurethane foams modified with natural lignocellulosic fillers, where increased open-cell content and filler hydrophilicity significantly intensified water uptake behavior [34,45].

### 4.5. Flammability

**Figure 11:**
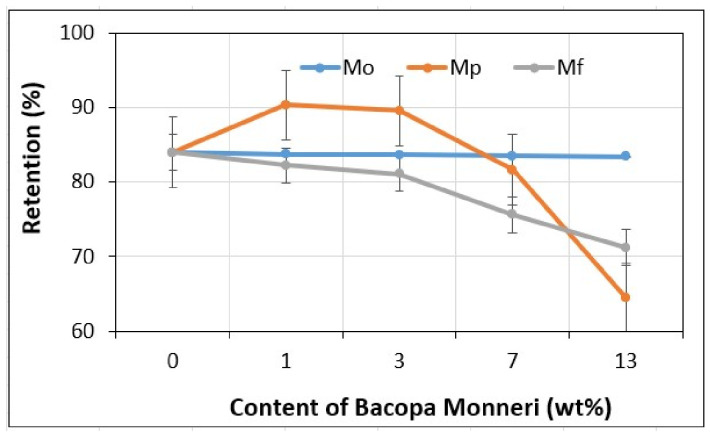
Dependence of retention on *Bacopa monnieri* content in foam.

**Figure 12:**
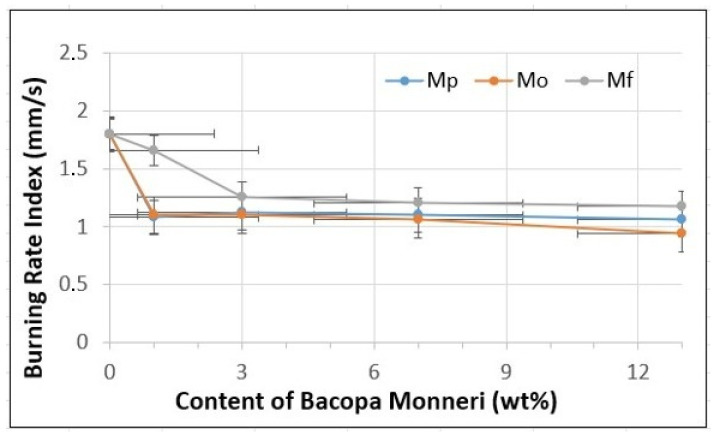
Dependence of burning rate index on *Bacopa monnieri* content in foam.

**Figure 13:**
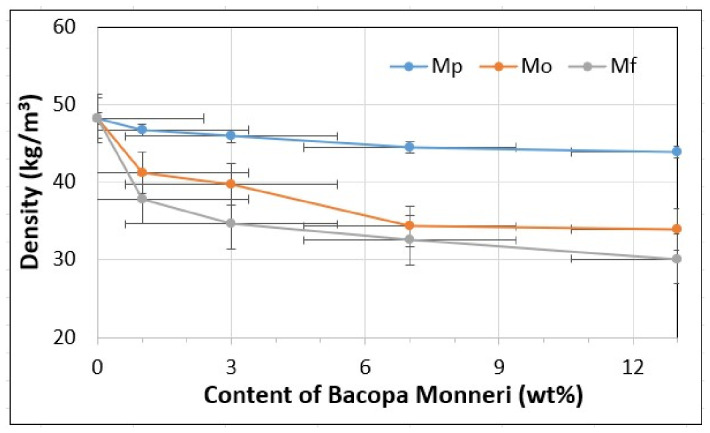
Dependence of density on *Bacopa monnieri* content in foam.

The fire-related behavior of RPU/PIR foams modified with different forms of *Bacopa monnieri* was evaluated based on retention after combustion (Figure 11) and the burning rate index (Figure 12). Both parameters provide insight into the flammability and fire resistance of the materials. Retention, defined as the residual mass after combustion, reflects the material’s ability to form a stable char layer during burning and is therefore directly related to its fire resistance. The error bars presented in Figure 12 and Figure 13 represent the standard deviations of the measurements and indicate the reproducibility of the observed trends.

For foams modified with powdered Bacopa monnieri (Mp), an initial increase in retention was observed, reaching approximately 90% at 1 wt% and remaining high at 3 wt%. However, with a further increase in filler content, a noticeable decrease occurred, dropping to approximately 64% at 13 wt%. These results suggest that low concentrations of the powder may enhance char formation and improve fire resistance, whereas higher contents may lead to structural degradation and reduced residue formation.

In the case of Mo-modified foams, retention remained relatively stable across the entire composition range (~83–84%). This indicates that this filler form does not substantially alter combustion residue formation and provides relatively consistent fire performance.

Foams containing spent residues (Mf) exhibited a gradual decrease in retention with increasing filler content, reaching approximately 71% at 13 wt%. This behavior suggests a moderate reduction in fire resistance, likely associated with the partial decomposition of lignocellulosic components during combustion.

The burning rate index provides direct information on flammability and flame propagation. A decrease in this parameter indicates improved fire performance. For Mp-modified foams, a reduction in burning rate was observed at low filler contents, decreasing from approximately 1.8 mm/s to ~1.1 mm/s at 1 wt%, followed by a slight additional decrease at higher contents. This behavior suggests suppression of flame propagation, most likely due to the formation of a protective char layer.

In the case of Mf-modified foams, the burning rate index decreased moderately and stabilized at higher filler contents (~1.15 mm/s), indicating a limited but consistent improvement in flammability behavior.

The combined analysis of retention and burning rate suggests that the form and content of Bacopa monnieri influence the fire performance of RPU/PIR foams. Among the investigated systems, the powdered form (Mp) showed the most pronounced reduction in burning rate at low filler concentrations. However, excessive filler loading was associated with lower residue formation. The tea form (Mo) ensured relatively stable fire performance across all compositions, while spent residues (Mf) provided moderate improvements despite a gradual decrease in retention.

### 4.6. Density and Compressive Strength

The compressive strength of foams depends on their density. Density, in turn, depends on the cell size and the content of open/closed cells, which are influenced by the surfactant, as well as the type of filler used. The smaller and more uniform the cells, the higher the strength. Uniform cells are formed as a result of the action of effective surfactants.

The results show that the lowest density (37.8–30.0 Kg/m^3^) was observed for foams containing *Bacopa monnieri* in the form of plant residues (Mf series foams) (Figure 13). At the same time, these foams exhibited the lowest compressive strength (342–634 KPa) (Figure 15). The highest density (44–48 Kg/m^3^) (and thus the highest compressive strength 510–733 KPa) was found in foams containing powdered, original powder *Bacopa monnieri* (Mp). The Mp foams (containing the unbrewed tea form of the plant) showed intermediate values for density and compressive strength.

**Figure 14:**
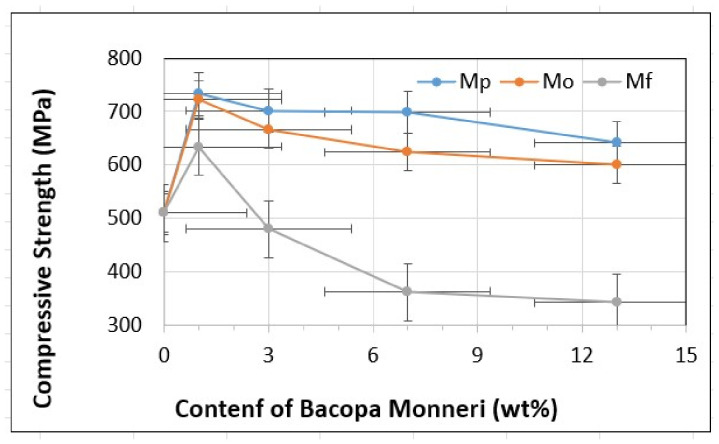
Dependence of compressive strength on *Bacopa monnieri* content in foam.

The compressive strength of RPU/PIR foams is directly correlated with density, which is governed by the cellular structure (Figure 14). Foams containing powdered *Bacopa monnieri* (Mp) exhibit the highest density and compressive strength, indicating the formation of a more uniform and compact cell structure, whereas foams with spent residues (Mf) show the lowest values due to the development of larger and less homogeneous cells. The form of the *Bacopa monnieri* filler significantly influences foam morphology and mechanical performance, with Mf promoting reduced density and mechanical strength, Mp resulting in intermediate properties, and Mp providing the most favorable structural conditions for enhancing compressive strength.

### 4.7. Brittleness

**Figure 15:**
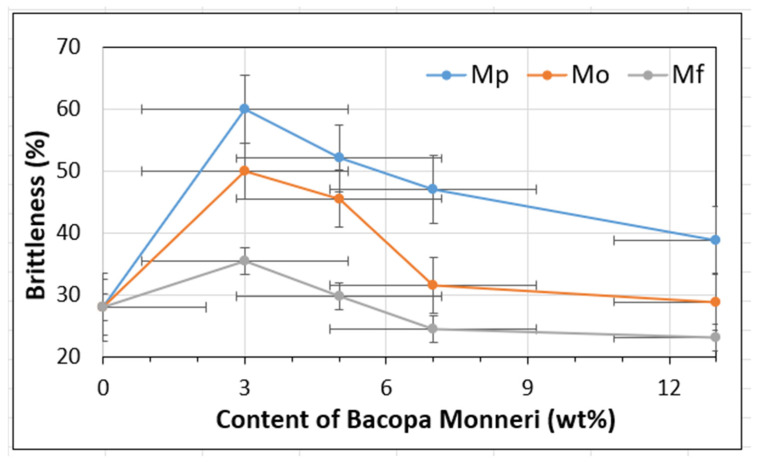
Dependence of *Bacopa monnieri* content in the foam on brittleness.

The brittleness of RPU/PIR foams is strongly influenced by both the content and form of *Bacopa monnieri*. The highest brittleness was observed at the 3 wt% filler content, reaching 59.9% for Mp, 49.9% for Mo, and 35.4% for Mf, indicating that this composition promotes the greatest structural heterogeneity and stress concentration within the polymer matrix (Figure 15). At a moderate filler loading (3 wt%), the filler particles are likely insufficient to form a reinforcing network, while simultaneously acting as localized defects disrupting the continuity of the foam structure. This promotes crack initiation and brittle fracture under mechanical stress.

With a further increase in filler content, brittleness decreased significantly, in some cases reaching values lower than that of the reference foam (M0), particularly for Mo13 and Mf13. This behavior suggests improved structural integration and more efficient stress redistribution at higher filler loadings. The increased amount of lignocellulosic filler may facilitate energy dissipation through enhanced filler–matrix interactions and partial stabilization of the cellular structure. The lowest brittleness was observed for foams containing spent residues (Mf), while the highest values were recorded for foams with powdered filler (Mp). This variation is attributed to differences in filler morphology and chemical composition, which affect dispersion, interfacial adhesion, and the degree of structural heterogeneity within the RPU/PIR matrix.

### 4.8. Thermal Properties

Thermal degradation of polyurethane foams proceeds through multistep decomposition processes, typically occurring within the range of 200–350 °C due to the dissociation of urethane and isocyanurate bonds [35,36]. Environmental factors such as UV radiation, humidity, and temperature may induce preliminary structural changes, including chain scission, oxidation, and rearrangement reactions, which subsequently affect the thermal response of the material [46,47]. Moreover, the incorporation of natural fillers may alter degradation kinetics, promote char formation, and increase the structural heterogeneity of polyurethane systems [48,49]. The degradation mechanisms of polyurethane materials under different thermal conditions were previously described by Shufen et al. [50].

The thermal stability of RPU/PIR foams modified with different forms of *Bacopa monnieri* (powder—Mp, tea—Mo, and post-extraction residues—Mf) was evaluated using thermogravimetric analysis (TGA) and differential scanning calorimetry (DSC). The obtained results indicate that both the additive form and filler loading significantly influence the degradation pathway and thermal stability of the foams [51,52].

#### 4.8.1. TGA

**Figure 16:**
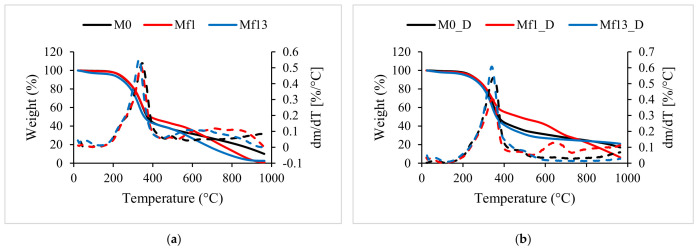
Dependence of *Bacopa monnieri* content in the non-degraded foams (**a**) and degraded foams (**b**) on TGA.

Based on the TG and DTG curves (Figure 16), all foams exhibited a characteristic three-stage degradation profile typical of RPU/PIR materials. The thermal degradation parameters, including T_5_%, T_10_%, T_20_%, T_50_%, and char residue (R%), are summarized in Table 4 and Table 5 for non-degraded and degraded foams, respectively. The maximum degradation temperatures (T_max_) and corresponding mass losses (Δm) obtained from DTG analysis are presented in Table 6 and Table 7.

For non-degraded foams, the incorporation of *Bacopa monnieri* affected thermal stability depending on both the filler form and concentration. Low and moderate filler contents generally improved the initial thermal resistance, particularly in the Mp7 and Mo1 systems, which exhibited higher T_5_% values compared to the reference foam (Table 4). In contrast, excessive filler loading (13 wt%) reduced thermal stability, especially in the Mf13 system, indicating increased structural heterogeneity and earlier degradation initiation.

The temperature corresponding to 50% mass loss (T_50_%) further confirmed that low filler contents enhanced resistance to bulk thermal decomposition. The highest T_50_% values were observed for the Mp1, Mp7, and Mo1 systems. However, increasing the filler content to 13 wt% resulted in lower thermal stability. These observations suggest that moderate amounts of *Bacopa monnieri* may contribute to the formation of more thermally stable structures, whereas excessive additive incorporation may weaken the continuity of the polymer matrix.

The degradation process occurred in three main stages, as confirmed by DTG analysis (Table 6). The first stage (~330–345 °C) was associated with urethane bond decomposition and represented the dominant mass-loss step. Modified foams exhibited lower Δm_1_ values than the reference sample, indicating partial redistribution of degradation processes and a greater contribution of subsequent degradation stages. The second stage (~480–520 °C) corresponded to the decomposition of polyisocyanurate structures and aromatic segments. In several modified systems, Tmax_2_ shifted toward higher temperatures, suggesting improved stability of thermally resistant domains. The third stage (~660–830 °C) was related to char transformation and stabilization of carbonaceous residues. Increased Tmax_3_ values, particularly for the Mo13 system, indicated enhanced thermal stability of the char layer formed in foams containing lignocellulosic components.

The char residue strongly depended on filler concentration and form. Moderate filler contents (3–7 wt%) promoted carbonization and increased residue yield, particularly in the Mo7, Mp3, and Mf7 systems. This behavior suggests that natural filler components facilitate char formation and reduce volatilization during high-temperature degradation.

Environmental degradation significantly influenced the thermal decomposition behavior of the foams (Table 6 and Table 7). In degraded samples, T_5_% values remained comparable to or slightly higher than those of the corresponding non-degraded systems at low filler contents, which may indicate partial structural rearrangement occurring during aging. However, samples containing 13 wt% filler exhibited a noticeable decrease in T_5_%, suggesting that excessive additive loading accelerated structural deterioration after environmental exposure.

More pronounced differences were observed for T_50_% and char residue values. Degraded foams generally exhibited higher T_50_% values than non-degraded materials, particularly for the Mo1_D, Mf1_D, and Mp1_D systems. Simultaneously, increased residue yields were observed after degradation, especially for the Mo3_D, Mp7_D, and Mp3_D foams. In addition, lower Δm_1_ values recorded for degraded systems suggest that partial decomposition of the most labile structures may have already occurred during environmental aging.

Overall, the results demonstrate that environmental degradation altered the degradation pathway of RPU/PIR foams by promoting preliminary chain scission, structural rearrangement, and enhanced carbonization. The observed increase in T_50_% and residue yield after degradation may also be associated with preferential degradation of low-molecular-weight fragments, oxidation-induced aromatization, and the formation of more thermally stable carbonaceous structures. Nevertheless, direct confirmation of a post-curing mechanism would require additional analyses, such as FTIR or DMA measurements.

To facilitate comparison between non-degraded and degraded foams, the most significant thermal stability changes after environmental degradation are summarized in Table 8.

#### 4.8.2. DSC

**Figure 17:**
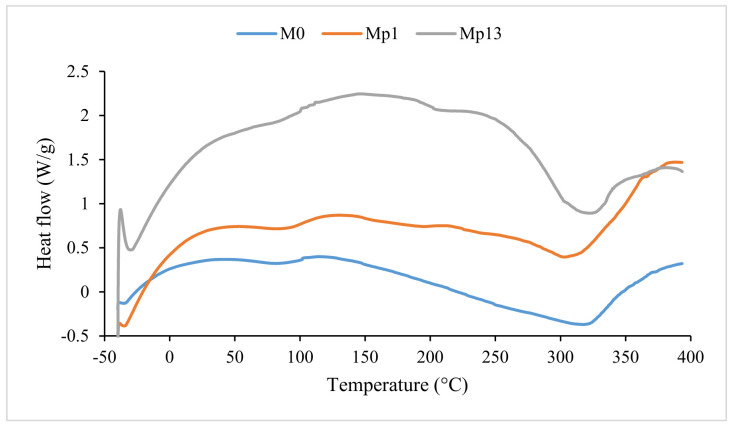
DSC of non-degraded foams: without filler (M0) and with extreme filler contents of 1 wt% (Mp1) and 13 wt% (Mp13).

**Figure 18:**
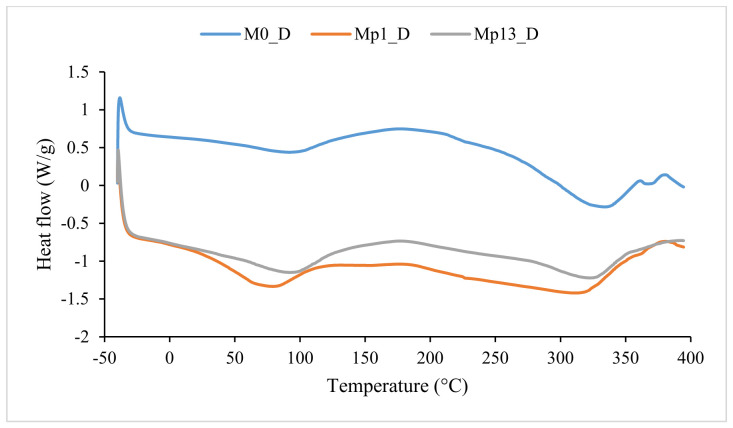
DSC of degraded foams: M0_D, Mp1_D, and Mp13_D.

**Figure 19:**
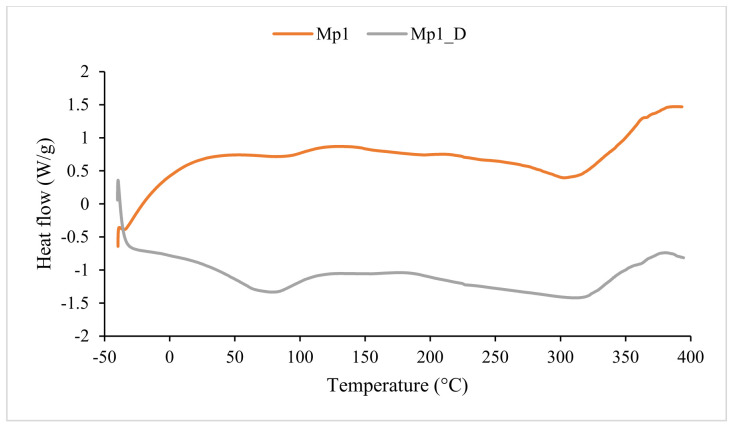
DSC: comparison of the non-degraded foam (Mp1) with the degraded foam (Mp1_D).

**Figure 20:**
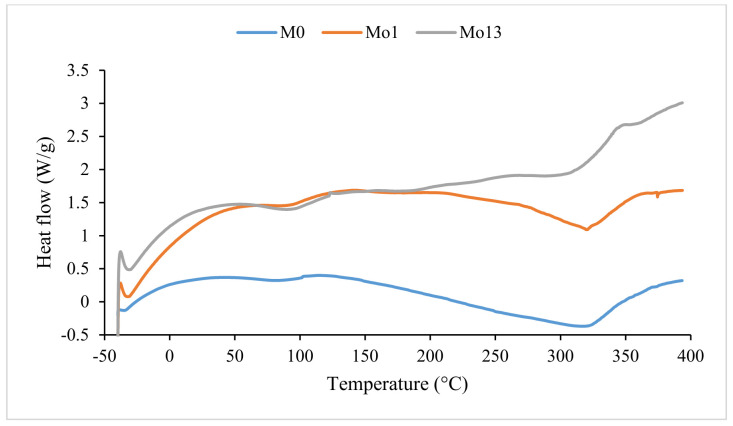
DSC of non-degraded foams: M0, Mo1 and Mo13.

**Figure 21:**
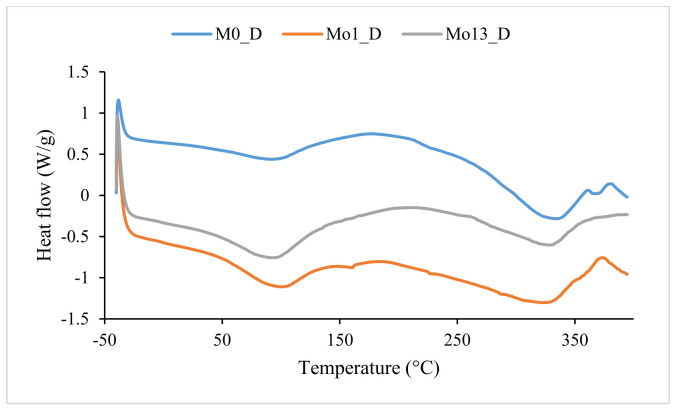
DSC of degraded foams: M0_D, Mo1_D, and Mo13_D.

**Figure 22:**
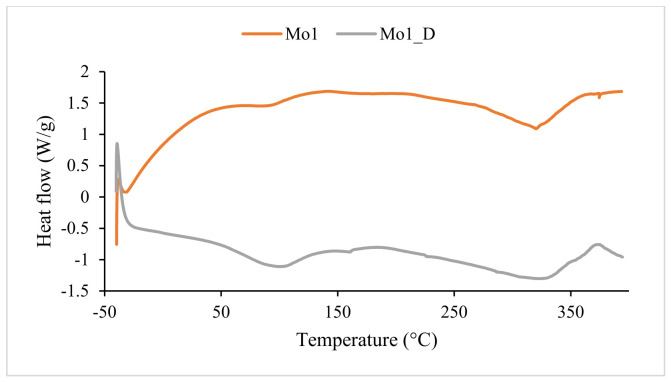
DSC of non-degraded foam Mo1 and degraded Mo1_D.

**Figure 23:**
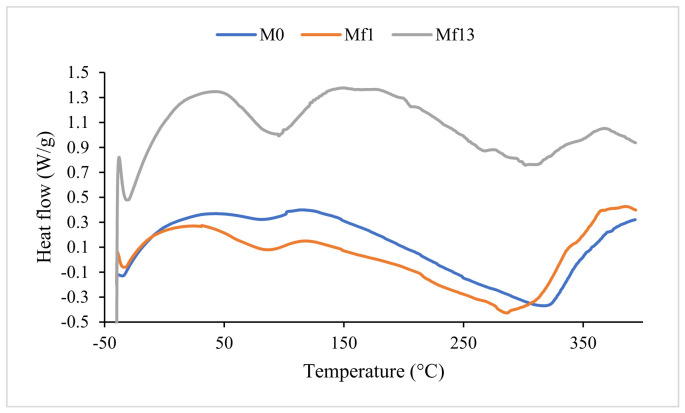
DSC non-degraded foams (M0) and foams containing residues (filler derived from *Bacopa monnieri* tea) (Mf1 and Mf13).

**Figure 24:**
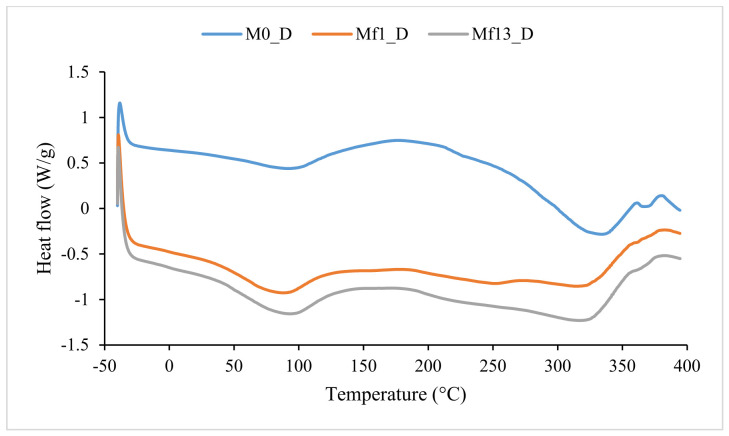
DSC of degraded foams: M0_D, Mf1_D, and Mf13_D.

**Figure 25:**
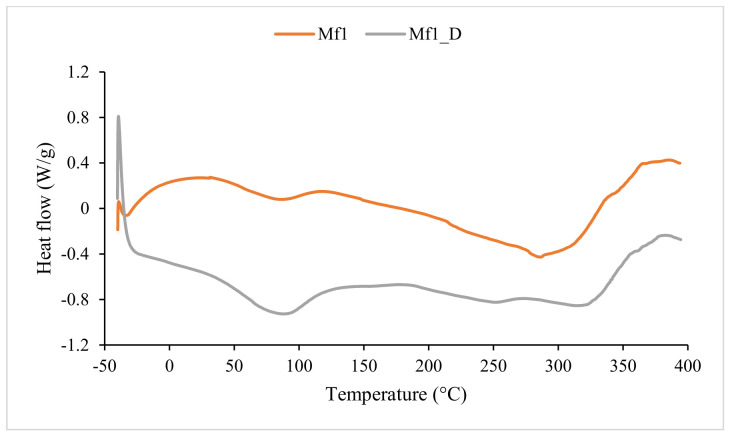
DSC of foam non-degraded Mf1 and degraded Mf1_D.

The DSC analysis confirmed that both the form and content of *Bacopa monnieri* significantly affected the thermal transitions and degradation behavior of RPU/PIR foams (Figure 17, Figure 18, Figure 19, Figure 20, Figure 21, Figure 22, Figure 23, Figure 24 and Figure 25). In agreement with the TGA results, the incorporation of plant-based additives increased the structural heterogeneity of the polyurethane systems and promoted multi-stage thermal decomposition. Similar effects were previously reported for lignocellulosic polymer composites, where natural fillers broaden thermal transitions and intensify overlapping degradation processes due to non-uniform heat distribution within the material [35,48,53].

For non-degraded foams, the DSC curves showed that low additive contents caused only minor changes in the thermal response compared to the reference foam. More pronounced differences were observed at higher filler loadings, particularly for Mo13 and Mf13 systems, which exhibited broader thermal transitions and increased heat-flow intensity. These effects indicate the enhanced heterogeneity of the polymer matrix and the presence of additional thermally active biomass-derived components.

The main thermal transitions observed in the DSC curves correspond well to the degradation stages identified in TGA. The temperature range of approximately 200–350 °C, associated in TGA with the decomposition of urethane and polyisocyanurate structures, was characterized in DSC by pronounced endothermic effects. Modified foams generally exhibited broader and more complex endothermic peaks than the reference system, confirming that the addition of *Bacopa monnieri* altered the degradation pathway from a relatively homogeneous process toward a multi-stage decomposition mechanism.

Among the investigated systems, foams containing the powder form of the additive (Mp) exhibited the highest thermal reactivity, particularly at low additive contents. The Mp1 sample showed the most intense endothermic effects in the main degradation region, indicating increased susceptibility to thermal decomposition. This observation is consistent with the TGA results, where lower Δm_1_ values and changes in Tmax suggested redistribution of degradation processes and earlier decomposition of labile structures. Increasing the additive content to 13 wt% reduced the intensity of DSC effects, suggesting partial stabilization of the polymer matrix.

The tea-derived additive (Mo) produced a different thermal response. The DSC curves of Mo-modified foams showed broader transitions and increased heat-flow intensity over a wide temperature range, particularly above 300 °C. These effects correspond to secondary degradation processes and char formation, which were also confirmed by the increased char residue observed in TGA. The presence of moisture and lignocellulosic structures in the Mo systems contributed to overlapping thermal effects associated with water evaporation, biomass decomposition, and structural rearrangement. The strongest stabilization effect after degradation was observed for the Mo13_D system, which exhibited reduced intensity of endothermic transitions and improved char stability.

Foams containing extraction residues (Mf) exhibited the most dispersed degradation behavior. Compared to Mp and Mo systems, the DSC curves of Mf foams were smoother and less intensive, indicating lower thermal reactivity and a more structural role of the filler. This observation correlates with the TGA results, where Mf systems showed enhanced carbonization and relatively stable high-temperature degradation behavior. The lignocellulosic character of the residue filler promoted gradual decomposition and char formation rather than rapid thermal degradation.

Environmental degradation significantly modified the DSC response of all investigated foams. Degraded systems generally exhibited stronger and broader endothermic effects than non-degraded counterparts, indicating increased heterogeneity and the presence of partially degraded structures generated during UV, humidity, and temperature exposure. These observations are consistent with TGA results, where degraded foams showed lower Δm_1_ values and increased T_50_% and char residue values, suggesting partial pre-degradation followed by structural rearrangement and enhanced carbonization during thermal analysis.

The most significant differences between degraded and non-degraded foams were observed in the 200–350 °C range, corresponding to the main degradation stage of polyurethane structures. Degraded foams exhibited intensified endothermic effects and broader thermal transitions, confirming that environmental aging promoted chain scission and the formation of thermally unstable fragments. Simultaneously, several degraded systems, particularly Mo_D and Mf_D foams, exhibited improved stability at higher temperatures, which corresponds well to the increased char formation observed in TGA.

Overall, the combined DSC and TGA analyses demonstrate that environmental degradation modifies the thermal degradation mechanism of RPU/PIR foams from a relatively homogeneous decomposition process toward a more complex pathway involving preliminary degradation, structural rearrangement, and intensified carbonization. The extent of these effects strongly depended on both the form and concentration of the *Bacopa monnieri* additive. Moderate filler contents promoted char formation and improved final-stage thermal stability, whereas excessive filler loading increased structural heterogeneity and accelerated initial degradation processes.

Początek formularza

Dół formularza

Dół formularza

The relatively small variations in Tmax values and the absence of substantial changes in the intensity of the main thermal effects suggest that *Bacopa monnieri* does not fundamentally alter the primary degradation chemistry of the RPU/PIR matrix. The decomposition of urethane and isocyanurate structures remains the dominant thermal process, while the plant-based additive acts mainly as a physical modifier affecting heat transfer, structural heterogeneity, and char formation. The lignocellulosic components of *Bacopa monnieri* promote carbonization and broaden the degradation stages rather than introducing new highly energetic degradation pathways. Consequently, the thermal decomposition mechanism is redistributed over a wider temperature range, leading to broader DSC transitions and moderate T_max_ shifts without substantial changes in the overall intensity of the thermal effects.

## 5. Conclusions

The obtained results demonstrated that the form of *Bacopa monnieri* significantly influences the physicochemical, mechanical, thermal, and flammability properties of rigid polyurethane–polyisocyanurate (RPU/PIR) foams. Polyphenols present in *Bacopa monnieri* may provide protection against ultraviolet radiation, whereas the incorporation of plant-derived fillers can simultaneously accelerate degradation processes due to the presence of cellulose structures. This phenomenon may be advantageous from the perspective of post-use environmental degradation of polymeric materials.

The mechanical performance of the foams was strongly dependent on their apparent density, which in turn was influenced by the type of filler used. The lowest density and compressive strength values were observed for foams containing spent *Bacopa monnieri* residues (Mf series). In contrast, the incorporation of powdered *Bacopa monnieri* (Mp series) resulted in the highest density and compressive strength. At the same time, the Mf foams exhibited reduced brittleness compared to the reference foam, indicating improved resistance to brittle fracture.

The results for water absorption and absorptivity confirmed that the form of the natural filler plays a crucial role in controlling the moisture-related behavior of RPU/PIR foams and should therefore be selected according to the intended application. Flammability analysis demonstrated that low to moderate filler contents provided the most favorable balance between a reduced burning rate and effective char formation.

Thermal analysis showed that the addition of *Bacopa monnieri* powder (Mp), particularly at higher filler contents, improved the thermal stability of RPU/PIR foams, suggesting favorable interactions between the filler and the polyurethane matrix. In contrast, the incorporation of *Bacopa monnieri* tea (Mo) led to a transition from a relatively homogeneous degradation mechanism to a more complex multi-stage degradation process accompanied by intensified charring phenomena, especially at higher additive concentrations. The application of spent residues (Mf) reduced the thermal reactivity of the foams and promoted a more dispersed degradation process compared with the unprocessed material, most likely due to the extraction of low-molecular-weight compounds during the brewing process.

Furthermore, the addition of *Bacopa monnieri* altered the degradation mechanism of RPU/PIR foams subjected to environmental aging. Aging processes induced chain scission and the formation of reactive functional groups, followed by structural reorganization involving partial crosslinking, formation of more aromatic structures, and intensified char formation, ultimately contributing to improved residual thermal stability. The incorporation of *Bacopa monnieri* tea (Mo) resulted in greater system heterogeneity and a more complex degradation pathway compared with the powdered form (Mp), which exerted a stronger influence on degradation kinetics and DSC transition intensity. At the highest filler content (13 wt%), all modified systems exhibited a tendency toward improved thermal stability; however, the mechanism responsible for this effect depended strongly on the form of the applied *Bacopa monnieri* filler.

## Figures and Tables

**Table 1:** Methods.

Name	Description	Standard
Apparent density	Five 50 ± 1 mm cube samples	ISO 845:2006
Compressive strength	Five 50 ± 1 mm cube samples; Instron 5544	ISO 844:2021
Microscope: VHX-X1	Keyence Corporation, Osaka, Japan, 200 magnification	-
FTIR	Range: 4000–400 cm^−1^, maximum resolution < 0.4 cm^−1^; Ncolet iS10, Thermo Fisher Scientific, Waltham, MA, USA	-
Flammability test: vertical Butler test	Vertical column measuring 300 × 57 × 54 mm with a movable glass front; five samples: 150 × 19 × 19 mm, weighed to the nearest 0.001 mm; propane–butane was applied for 10 s	ASTM D3014
Horizontal test	Five samples: 150 × 50 × 13 mm, flame applied for 60 s	PN-78 C-05012 (PN-C-05012-12:1978)
Absorbability, water absorption	Five samples: 150 × 150 × 25 mm, measured after immersion in distilled water (24 h)	ISO 280 2896:2001
TGA	Q500 thermobalance, TA Instruments, New Castle, DE, USA; sample: weight 21 mg, temperature: from 0 to 1000 °C	
DSC	Q200 (TA Instruments, New Castle, DE, USA); apparatus working range: from −90 to +400 °C in nitrogen	
Degradation	Climatic chamber (DYCOMETAL CCK, model CCK-40/300 NG, Es-tor Sp. z o.o., Poznan, Poland), temperature of 50 °C, relative humidity of 70%, UV radiation of 320.86 W/m^2^, time: 3 weeks	

**Table 2:** Foam processing parameters.

Foam	Cream Time (s)	String Time (s)	Tack Free Time (s)	Free Rise Time (s)	T_max_ (°C)
M0	10	28	31	40	171
Mp1	15	25	28	31	178
Mp3	15	26	29	33	178
Mp7	17	40	44	46	174
Mp13	20	42	52	54	174
Mo1	15	31	32	36	174
Mo3	13	25	27	39	178
Mo7	17	28	32	38	178
Mo13	21	51	56	56	178
Mf1	11	21	23	24	176
Mf3	11	22	24	25	176
Mf7	12	24	27	32	176
Mf13	13	40	47	48	176

**Table 3:** Microscopic analysis results.

Foam	Cell Height (μm)	Cell Width (μm)	Anisotropy (-)
M0p	538	463	0.86
Mp1p	226	221	0.98
Mp3p	220	193	0.88
Mp7p	352	226	0.63
Mp13p	425	294	0.69
Mo1p	349	303	0.87
Mo3p	262	216	0.82
Mo7p	229	225	0.98
Mo13p	375	254	0.67
Mf1p	314	264	0.84
Mf3p	250	206	0.82
Mf7p	256	226	0.88
Mf13p	366	339	0.93
M0z	477	326	0.68
Mp1z	309	268	0.87
Mp3z	340	212	0.62
Mp7z	467	276	0.58
Mp13z	589	354	0.53
Mo1z	425	224	0.53
Mo3z	560	282	0.51
Mo7z	266	183	0.69
Mo13z	383	246	0.64
Mf1z	312	160	0.51
Mf3z	325	163	0.51
Mf7z	467	346	0.74
Mf13z	430	281	0.64
M0p_D	680	528	0.78
Mp1p_D	212	176	0.83
Mp3p_D	218	182	0.83
Mp7p_D	223	182	0.81
Mp13p_D	268	234	0.87
Mo1p_D	327	260	0.79
Mo3p_D	185	152	0.82
Mo7p_D	246	199	0.81
Mo13p_D	263	216	0.82
Mf1p_D	195	168	0.86
Mf3p_D	136	120	0.88
Mf7p_D	218	136	0.63
Mf13p_D	420	298	0.71
M0z_D	461	423	0.92
Mp1z_D	327	214	0.65
Mp3z_D	267	223	0.84
Mp7z_D	292	170	0.58
Mp13z_D	328	180	0.55
Mo1z_D	490	335	0.68
Mo3z_D	344	163	0.47
Mo7z_D	302	206	0.68
Mo13z_D	314	247	0.79
Mf1z_D	334	210	0.63
Mf3z_D	174	107	0.61
Mf7z_D	296	196	0.66
Mf13z_D	440	281	0.64

**Table 4:** Thermal stability parameters of non-degraded foams determined by TGA analysis (mean ± SD, n = 5).

Foam	T5% (°C)	T10% (°C)	T20% (°C)	T50% (°C)	R% (%)
M0	231.2 ± 1.4	262.3 ± 2.1	304.3 ± 1.8	371.9 ± 3.5	10.1 ± 0.4
Mp1	240.2 ± 2.0	271.1 ± 1.7	311.8 ± 2.2	425.4 ± 4.1	2.1 ± 0.2
Mp3	229.3 ± 1.6	259.0 ± 2.3	300.8 ± 2.0	362.8 ± 2.8	16.8 ± 0.5
Mp7	246.1 ± 2.2	274.5 ± 1.9	310.5 ± 2.4	426.0 ± 3.7	2.6 ± 0.3
Mp13	219.2 ± 1.5	249.9 ± 2.0	293.5 ± 1.7	358.2 ± 2.5	8.1 ± 0.4
Mo1	239.0 ± 1.9	270.2 ± 1.8	308.8 ± 2.1	396.0 ± 3.9	2.1 ± 0.2
Mo3	219.6 ± 1.7	248.9 ± 1.9	292.0 ± 1.8	359.6 ± 2.6	10.5 ± 0.5
Mo7	219.4 ± 1.8	249.9 ± 2.1	292.1 ± 1.9	354.5 ± 2.9	18.0 ± 0.6
Mo13	218.5 ± 1.6	251.5 ± 2.0	292.2 ± 1.8	359.3 ± 2.7	7.7 ± 0.4
Mf1	234.3 ± 1.5	265.0 ± 1.7	305.6 ± 2.0	387.6 ± 3.3	1.0 ± 0.1
Mf3	225.9 ± 1.7	256.1 ± 2.1	297.8 ± 1.9	363.1 ± 2.8	7.3 ± 0.3
Mf7	224.7 ± 1.8	256.6 ± 2.0	297.4 ± 1.8	357.1 ± 2.7	14.1 ± 0.5
Mf13	197.8 ± 1.9	242.5 ± 2.2	288.5 ± 2.1	356.9 ± 2.9	2.6 ± 0.2

**Table 5:** Thermal stability parameters of degraded foams determined by TGA analysis (mean ± SD, n = 5).

Foam	T5% (°C)	T10% (°C)	T20% (°C)	T50% (°C)	R% (%)
M0_D	236.5 ± 1.7	269.9 ± 2.1	308.8 ± 2.0	372.5 ± 3.6	17.0 ± 0.6
Mp1_D	236.3 ± 1.8	275.1 ± 2.2	318.3 ± 2.4	452.6 ± 4.5	19.5 ± 0.7
Mp3_D	228.1 ± 1.6	265.9 ± 2.0	310.5 ± 2.1	410.8 ± 3.9	24.8 ± 0.8
Mp7_D	239.5 ± 1.9	275.0 ± 2.1	313.2 ± 2.3	398.0 ± 3.7	27.6 ± 0.9
Mp13_D	209.2 ± 1.5	246.8 ± 1.9	294.5 ± 1.8	365.3 ± 3.1	24.2 ± 0.8
Mo1_D	234.9 ± 1.8	280.4 ± 2.3	324.4 ± 2.5	503.9 ± 5.0	13.0 ± 0.5
Mo3_D	231.8 ± 1.7	270.1 ± 2.0	314.0 ± 2.2	424.6 ± 4.1	28.7 ± 0.9
Mo7_D	220.5 ± 1.6	264.3 ± 2.1	310.5 ± 2.0	458.2 ± 4.6	7.9 ± 0.4
Mo13_D	203.5 ± 1.5	257.2 ± 2.0	306.3 ± 2.1	422.2 ± 4.0	22.8 ± 0.8
Mf1_D	233.1 ± 1.7	270.8 ± 2.1	313.6 ± 2.2	471.8 ± 4.7	6.2 ± 0.3
Mf3_D	218.0 ± 1.6	258.5 ± 2.0	304.7 ± 2.0	398.8 ± 3.8	19.4 ± 0.7
Mf7_D	217.3 ± 1.5	260.3 ± 2.1	304.6 ± 2.0	375.4 ± 3.5	8.8 ± 0.4
Mf13_D	208.5 ± 1.5	256.8 ± 2.0	301.8 ± 1.9	362.4 ± 3.2	21.0 ± 0.7

**Table 6:** Maximum degradation temperatures and mass losses of non-degraded foams obtained from DTG curves (mean ± SD, n = 5).

Foam	Tmax1 (°C)	Δm1 (%)	Tmax2 (°C)	Δm2 (%)	Tmax3 (°C)	Δm3 (%)
M0	346.3 ± 1.1	60.2 ± 1.5	488.7 ± 2.0	4.6 ± 0.3	765.3 ± 5.1	24.7 ± 1.0
Mp1	344.0 ± 1.5	45.8 ± 1.2	481.3 ± 1.9	10.3 ± 0.4	693.4 ± 4.3	41.2 ± 1.6
Mp3	336.3 ± 1.3	58.7 ± 1.4	522.0 ± 2.4	7.5 ± 0.4	744.4 ± 4.8	16.7 ± 0.9
Mp7	336.7 ± 1.4	47.0 ± 1.1	496.4 ± 2.1	8.3 ± 0.3	701.9 ± 4.5	42.6 ± 1.7
Mp13	329.9 ± 1.6	55.0 ± 1.3	513.6 ± 2.3	6.1 ± 0.3	751.8 ± 5.0	30.0 ± 1.3
Mo1	341.6 ± 1.3	47.8 ± 1.1	480.1 ± 2.0	9.5 ± 0.4	682.3 ± 4.2	39.8 ± 1.5
Mo3	338.0 ± 1.2	55.8 ± 1.4	519.1 ± 2.4	10.1 ± 0.5	767.3 ± 5.3	22.6 ± 1.0
Mo7	334.9 ± 1.5	58.4 ± 1.5	510.5 ± 2.2	11.1 ± 0.5	748.5 ± 5.0	11.7 ± 0.8
Mo13	329.2 ± 1.7	55.0 ± 1.3	514.4 ± 2.3	10.2 ± 0.4	827.8 ± 6.0	25.7 ± 1.2
Mf1	339.7 ± 1.3	49.4 ± 1.2	485.9 ± 2.1	9.1 ± 0.4	709.8 ± 4.4	39.8 ± 1.5
Mf3	334.1 ± 1.4	56.1 ± 1.4	494.7 ± 2.2	6.5 ± 0.3	664.1 ± 4.0	29.7 ± 1.2
Mf7	335.9 ± 1.5	58.5 ± 1.5	509.9 ± 2.3	9.3 ± 0.4	731.6 ± 4.7	17.3 ± 0.9
Mf13	330.9 ± 1.6	53.0 ± 1.2	482.2 ± 2.0	7.0 ± 0.3	693.1 ± 4.3	34.0 ± 1.4

**Table 7:** Maximum degradation temperatures and mass losses of degraded foams obtained from DTG curves (mean ± SD, n = 5).

Foam	Tmax1 (°C)	Δm1 (%)	Tmax2 (°C)	Δm2 (%)	Tmax3 (°C)	Δm3 (%)
M0_D	346.2 ± 1.3	53.8 ± 1.4	494.4 ± 2.2	11.9 ± 0.5	741.7 ± 4.8	16.5 ± 0.9
Mp1_D	349.3 ± 1.5	42.2 ± 1.1	497.8 ± 2.3	14.4 ± 0.6	758.7 ± 5.0	22.7 ± 1.0
Mp3_D	347.4 ± 1.4	45.8 ± 1.2	460.9 ± 2.0	14.5 ± 0.6	743.1 ± 4.7	13.8 ± 0.8
Mp7_D	339.4 ± 1.3	46.1 ± 1.2	462.3 ± 2.1	18.0 ± 0.7	761.9 ± 5.1	7.2 ± 0.4
Mp13_D	334.9 ± 1.5	50.2 ± 1.3	450.7 ± 1.9	16.5 ± 0.6	734.7 ± 4.6	7.6 ± 0.5
Mo1_D	349.4 ± 1.5	36.1 ± 1.0	493.5 ± 2.2	15.4 ± 0.6	657.7 ± 4.0	13.9 ± 0.8
Mo3_D	345.6 ± 1.4	42.5 ± 1.1	482.6 ± 2.1	19.7 ± 0.7	790.1 ± 5.4	8.4 ± 0.5
Mo7_D	337.8 ± 1.3	42.9 ± 1.2	481.6 ± 2.1	10.8 ± 0.5	752.5 ± 4.9	37.0 ± 1.5
Mo13_D	335.7 ± 1.4	42.7 ± 1.1	490.4 ± 2.2	12.7 ± 0.5	763.9 ± 5.0	19.5 ± 0.9
Mf1_D	342.1 ± 1.3	41.6 ± 1.1	486.4 ± 2.1	11.2 ± 0.5	635.2 ± 3.9	18.1 ± 0.9
Mf3_D	342.2 ± 1.4	46.8 ± 1.2	470.1 ± 2.0	13.7 ± 0.6	683.9 ± 4.2	18.6 ± 0.9
Mf7_D	340.7 ± 1.3	49.6 ± 1.3	446.5 ± 1.8	15.7 ± 0.6	741.7 ± 4.8	24.3 ± 1.1
Mf13_D	339.6 ± 1.4	54.7 ± 1.4	496.3 ± 2.3	15.6 ± 0.6	753.2 ± 4.9	6.6 ± 0.4

**Table 8:** Comparative summary of thermal stability changes after environmental degradation.

Foam System	Change in T_5_% After Degradation	Change in T_50_% After Degradation	Change in Char Residue (R%)	Main Observation
M0	Slight increase	Comparable	Increase	Structural rearrangement after aging
Mp (1–7 wt%)	Stable/slight increase	Significant increase	Strong increase	improved carbonization and delayed bulk degradation
Mp13	Decrease	Slight increase	Increase	Excessive filler content reduced structural stability
Mo1	Stable	Very strong increase	Moderate increase	Highest thermal stability after degradation
Mo3–13	Decrease with filler content	Increase	Strong increase	Enhanced char formation
Mf systems	Gradual decrease	Moderate increase	Variable	Lower resistance at high filler contents

## Data Availability

The original contributions presented in this study are included in the article. Further inquiries can be directed to the corresponding author.

## Abbreviations

The following abbreviations are used in this manuscript:

BM	*Bacopa monnieri*
Mp	RPU/PIR with powder of BM
Mo	RPU/PIR with powder tea of BM
Mf	RPU/PIR with spent residues of BM
